# Online measurement of temperature and relative humidity as marker tools for quality changes in onion bulbs during storage

**DOI:** 10.1371/journal.pone.0210577

**Published:** 2019-01-17

**Authors:** Md. Nahidul Islam, Aimei Wang, Jakob Skov Pedersen, Jørn Nygaard Sørensen, Oliver Körner, Merete Edelenbos

**Affiliations:** 1 Department of Food Science, Aarhus University, Aarslev, Denmark; 2 Danish Technological Institute, Taastrup, Denmark; 3 Leibniz Institute of Vegetable and Ornamental Crops, Grossbeeren, Germany; Northwest Agriculture and Forestry University, CHINA

## Abstract

A long shelf life of onions (*Allium cepa* L.) is of high importance in the onion industry. Onions are dried and stored in large wooden boxes that are difficult to access. Monitoring temperature and relative humidity during these processes is challenging. Moreover, quality may change in storage without being noticed. Therefore, there is a need to find alternative methods for monitoring and controlling the drying and storage processes of onions and to identify early changes in quality during storage. The potential use of online measurements of temperature and relative humidity (RH) in the vicinity of onions was evaluated during drying and long-term storage of six onion batches (four cultivars and three selections of one of the cultivars) in commercial storage. The batches varied in bulb weight, dry matter content, firmness and disease incidence. The dry matter content and firmness decreased during storage, while the respiration rate and incidences of individual and total disease increased. Two of the batches had low storability with high disease incidences and high average temperatures and variations in the RH. The results showed that tracking the temperature and RH in the vicinity of the onions is a promising tool for improving the drying and storage processes in commercial storage and for identifying onion batches with reduced storability early in storage.

## Introduction

A long shelf life of onions (*Allium cepa* L.) is of high importance in the onion industry. Several factors determine the shelf life and storability of onions, e.g., the cultivar, growing conditions, harshness in harvesting, postharvest drying and storage, and storage duration [1]. In wet climates as in North Europe, onions are lifted, naturally cured in the field to dry the outer surface and neck so it forms a sealed, yellow tunic of dried and death scales, harvested, artificially dried in storage rooms to further cure the bulb, and stored at low temperature for prolonged periods to reduce metabolic activity and decay.

Onions are living products that respire in storage [1] and use carbohydrates and other constituents as substrates in respiration, and bulbs eventually become senescent and deteriorate. The storage temperature influences the metabolic turnover in respiration. However, rooting, sprouting, and developing disease may also influence respiration [2–4] and it may lead to considerable food losses and waste [5] if bulbs are not sold before high losses occur.

The most severe diseases of onions are basal rot (*Fusarium oxysporum*), blue mold (*Penicillium* spp.), neck rot (*Botrytis allii*/*aclada*), gray mold (*Botrytis cinerea*), sour skin (*Burkholderia cepacia*) and slippery skin (*Burkholderia gladioli* pv. *alliicola*) [4, 6]. Basal rot is a disease that infects bulbs in the soil through the roots and basal plate during growth [4, 7], while blue mold is a secondary pathogen that infects through wounds formed during harvesting and presorting or through already-diseased tissue [4]. In contrast, neck rot, gray mold, sour skin, and slippery skin infect through the neck and occur if the bulbs are not properly dried and sealed during curing [1]. In Denmark, onions are cured artificially at 20 °C (conventional onions) for prolonged times or at 35 °C (organic onions) for two weeks to seal the neck and tunic to prevent diseases from developing during cold storage. However, in other countries, the drying temperature during curing and the drying duration may be different, depending on how well the bulbs are naturally cured in the field at harvest [8–10]. Following curing, the bulbs are stored at low temperature (approximately 0 °C) and low relative humidity (RH) (65–75%) to prevent microbial growth, sprouting, and rooting [1]. Nevertheless, bulbs may still sprout and spoil in storage due to physiological changes and disease development, especially in organic production [3].

Onions are stored in bulk storage or in large wooden boxes. Thus, monitoring bulb quality in storage is challenging, as bulbs are difficult to access. Moreover, diseases may develop in storage without being noticed, as most onion diseases develop inside the bulb [3]. Therefore, there is a need for better management tools in the onion industry for the continuous monitoring of onion quality in storage. It has been reported that increased respiration is a common and prominent feature of infected plant tissue [11]. Similar results were observed by Wang, Casadei [12], who found that diseased onion bulbs had higher respiration rates than healthy bulbs. Respiratory heat and water vapor are released during respiration, and ideally, this heat and water vapor is removed by refrigeration and ventilation to maintain a constant temperature and relative humidity in storage. However, it is not known if measurement of temperature and relative humidity in the vicinity of onions is a possible management tool for monitoring bulb quality in storage. This study was designed to investigate the microclimate in the vicinity of different batches of onions in storage and to relate these data with information on quality. We hypothesize that diseased onions release more respiratory heat and water vapor than healthy onions and thus have higher average temperatures and RH in storage.

## Materials and methods

### Onion batches

**Fig 1:**
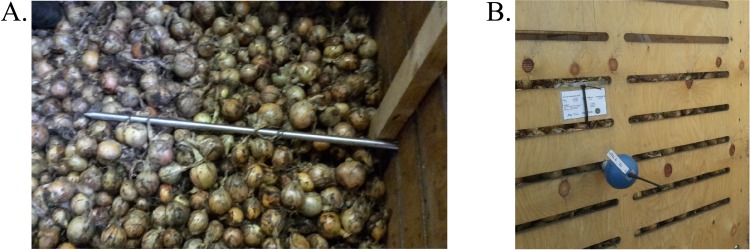
Placement of the Hortisens spear in a two-ton box with onions. **Inside view of the box at the time of filling with middle sensor 8 cm away from the tip and edge sensor next to the wooden side of the box (A). Outside view with transmitter (blue ball) and black antenna (B).** Photos: Jacob Skov Pedersen, Technological Institute.

Six batches of organic onions were selected to represent different cultivars (‘Barito’, ‘Hylander’, ‘Hypark’, and ‘Summit’) and qualities (‘Summit’ from fields 246, 247 and 436) of yellow onions (*Allium cepa* L.) (Table 1). The onions were grown organically in 2015 by a commercial grower (Axel Månsson A/S, Brande, Denmark, LAT 56°) on sandy soil in different fields without the use of synthetic fertilizers and chemicals (inorganic NPK, pesticides, sprout inhibitors, etc.). ‘Barito’ and ‘Hylander’ were directly seeded, and ‘Hypark’ and ‘Summit’ were transplanted. The bulbs were lifted and windrowed at 80% top-fall, cured and surface-dried for 7 days, and then harvested. Small bulbs, stones and soil particles less than 38 mm were removed during harvest and the remaining crop loaded directly on to transport vehicles, except for batch 6, which was filled directly into two-ton wooden storage boxes (160 cm × 120 cm × 120 cm) from the hopper and then transported to the farm. The five batches were presorted at the farm before yard filling into the two-ton storage boxes. Weeds and other foreign materials, small bulbs, stones and soil particles (less than 38 mm) and large stones (more than 150 mm) were removed in presorting. Hortisens spears (Webstech, Kongskilde, Denmark) were inserted during the filling of the boxes (Fig 1) for online measurements of temperature and RH during artificial curing and drying, and storage [3]. The spears were 85 cm long and had temperature and RH sensors at 8 cm away and another at 78 cm away from the tip of the spear. The temperature and relative humidity readings of the spears were crosschecked against a calibrated MSR sensor (MSR Electronics GmBH, Switzerland) before use, and the readings were adjusted against the MSR sensor. The accuracy of the spears were ± 0.4 °C and ± 3% RH and the accuracy of the MSR sensors were ± 0.5 °C and ± 2% RH. The temperature in the middle of the box was recorded with the sensor closest to the tip of the spear, while the temperature at the edge of the box was recorded with the sensor furthest away from the tip. The spear had a transmitter with an antenna, which was placed outside of the box at the end of the spear (Fig 1). The data were transmitted through a base station installed in the storage room to a central server for online readings.

**Table 1:** Batches of onions for storage^a^.

Batch no.	Cultivar^b^	Field	Harvest date 2015	Days in curing^c^	Bulb weight^d^ (g)	Bulb size^e^ (mm)	Quality of onions in field at 80% top-fall
B1	Barito	287	3 October	22	130a^f^	67a^f^	Uneven bulb size
B2	Hylander	293	2 October	23	110b	63ab	
B3	Hypark	248B	29 September	26	95bc	62ab	
B4	Summit	246	27 September	28	110b	64ab	*Fusarium oxysporum* on bulbs
B5	Summit	247	30 September	25	91c	60ab	
B6	Summit^g^	436	11 September	44	84c	57b	*Botrytis spp*. on leaves

^a^ Small bulbs, stones and soil particles less than 38 mm were removed during harvest. Weeds and other foreign materials, small bulbs, stones and soil particles (less than 38 mm) and large stones (more than 150 mm) were removed in presorting, unless noted.

^b^ Cultivars were seeded (‘Barito’ and ‘Hylander’) or transplanted (‘Hypark’ and ‘Summit’).

^c^ Days in curing were from harvest to drying temperature reached 20 °C after drying at 35 °C.

^d^ Average bulb weight across the three sampling times.

^e^ Average bulb size was calculated as 13.2 * bulb weight^1/3^ according to Brewster [1].

^f^ Means followed by different letters within column are significant different at *P* = 0.05 according to Tukey’s honest significance difference test.

^g^ Stored without presorting.

### Drying and storage

The boxes with the six batches were placed in the same storage room on top of six columns with other onion boxes so that the bulbs were accessible throughout the storage. Three of the boxes were placed next to each other in one row, and the other three were opposite to these ones in another row. The room contained 220 boxes in 44 columns placed in two rows with a 120-cm alley in between, and there was an 80-cm distance to the boundary walls. A VACCTEC system (Frigortek Cooling Systems Aps, Vandel, Denmark), which used forced ventilation, monitored and controlled the temperature and relative humidity during the drying and storage. While the drying was waiting to start, the bulbs were cured with ventilated air between 18 °C and 24 °C until the room was filled with onions. Batch 1 was the last batch to be harvested, and the bulbs in this batch were thus only ventilated for a few hours before the start of drying, while the bulbs of batch 6 were ventilated for 22 days before the start of drying (Table 1). Thus, some batches were surface-dried for longer than others because they were harvested earlier (Table 1). During drying, the temperature was set to increase slowly to 35 °C for two weeks and then to decrease steeply to 18 °C within a few days. Following the drying, the temperature was slowly decreased to 0.5 °C to adapt the bulbs to cold storage.

### Storage microclimate

The temperature and RH were recorded every hour, and from these values, the average daily temperature and RH were calculated. A lag plot for RH was prepared to study the variation in the RH between two consecutive measurements (*lagplot* function in Python, v3.6.3, Python Software Foundation, Beaverton, Oregon). From the data at time *t* and time *t* + 1 (*t* is in hours), correlation coefficients were determined. Additionally, the vapor pressure deficit (*VPD*) in kPa [13] was calculated in the middle of the boxes using Eq 1

$$VPD\;\;\;\;\;=\;e_{s}-\;\;e_{a}$$ (1)

where saturated (*e*_*s*_) is calculated using Eq 2

$$e_{s}=\;0.6108\;\;\;e^{\left(17.27\;\times \;T_{air}/T_{air}+265.5\right)}$$ (2)

where *T*_*air*_ (°C) is the measured air temperature and *RH*(%) is the measured relative humidity at a given temperature, and the unsaturated air (*e*_*a*_) is calculated using Eq 3

$$e_{a}=\;RH/100\;\;e_{s}$$ (3)

To calculate the contribution of the respiratory heat to the average 24-hour storage temperature in the middle of the box, the daily temperature difference, Δ*T*, was calculated from the differences in temperature at the middle and edge of the box using Eq 4

$$\Delta T\;=\;\bar{T_{i}}\;-\;\bar{T_{e}}$$ (4)

where ${\bar{T}}_{i}$ is the average 24-hour temperature (°C) in the middle of the box, and $\bar{T_{e}}$ is the average 24-hour temperature (°C) at the edge of the box. If *ΔT* was negative, which may occur during periods of reduced cooling, the data points were removed. From the VPD and Δ*T* values, daily cumulative VPD and Δ*T* sums were calculated using Eq 5 in MATLAB (version 9.3.0, MathWorks, Natick, MA, USA).

$$y_{i}\;=\;\sum_{j=1}^{i}x_{j}$$ (5)

where, *y* is the cumulative value, *x* is either the daily VPD or Δ*T*, and *i* ∈ {1,2,…,number of days}

### Sampling of bulbs for quality evaluation

The bulbs were sampled in December 2015 when the storage temperature reached 0.5 °C and again after 60 days and 110 days of cold storage. Samples of the onions (120 kg) were randomly taken from each batch, brought to Aarhus University (Aarslev, Denmark), and manually sorted into bulbs below 40 mm, bulbs between 40 and 80 mm, and bulbs greater than 80 mm. From the 40–80 mm size group, 3 subsamples of 5 kg each were randomly taken for the assessment of rooting, sprouting and diseases. As the average bulb weight varied between batches (Table 1), the total number of bulbs in each subsample varied, between 128 bulbs in batch 1 and 180 bulbs in batch 6. The weight of the onions in each subsample was recorded at each sampling time, and from these values, the average bulb weight and bulb size [1] were calculated at each sampling time. Moreover, three representative samples of 1 kg healthy, nonsprouted, firm, and 40–80 mm bulbs were taken for determination of the respiration rate, firmness and dry matter content at each storage time.

At the end of storage, the remaining bulbs in the two-ton boxes were automatically sorted on a commercial grading machine and total weights were recorded (Sammo, Longobardi, Italy). The machine had different grading features, among those a laser scattering unit for determination of bulb size and bulb volume, a weighing unit for identifying stones and other foreign materials, and bulbs with a low weight-to-volume ratio, and a spectral near-infrared (NIR) unit for internal disease monitoring [6]. From these data, the total weight of usable, healthy and nonsprouted bulbs in each size category was determined.

### Respiration rate

The respiration rate of the healthy onions was determined in triplicate, as described by Islam [13], starting on the day after the sampling time. The onions were equilibrated to the storage temperature (18 °C), and three onions were incubated in a 1-L glass jar and closed with an airtight lid that had a hole with a septum for gas sampling. The gas composition was measured with a CheckMate 9900 Headspace Gas Analyzer (Dansensor A/S, Ringsted, Denmark) equipped with a zirconia sensor for O_2_ measurements and a dual-beam infrared sensor for CO_2_ measurements. The respiration rates for O_2_ and CO_2_, both in ml kg^-1^ h^-1^, were determined from the differences in the O_2_ and CO_2_ contents at the beginning and after 24 h of incubation at 18 °C [13]. As the maximum O_2_ and CO_2_ contents during incubation went down to 13% O_2_ and up to 8% CO_2_ it was not expected that this gas modification had any effect on the respiration rate of onion bulbs. Only the respiration rate for CO_2_ was reported, as the respiration rate for O_2_ gave similar results.

### Firmness and dry matter content

Firmness was determined in a compression test, as described by Coolong and Randle [14], and Islam, Nielsen [15] with slight modifications. A 10-mm-diameter probe (TA10) was mounted on a CT3 texture analyzer (Brookfield Engineering Laboratories, Inc., Middleboro, Massachusetts). The initial target value, trigger load and test speed were 30%, 0.07 N and 30 mm min^-1^, respectively. Three healthy onions were taken at each sampling. The outer dried scales were removed, and the first fully fleshy scale used. Three pieces of 2 × 4 cm each were taken at the equatorial region of the scale and measured at the convex side. The force necessary to penetrate the onion pieces, the thickness of the scale pieces, and the depth in mm at penetration were noted, and from these values, a firmness index (FI) was determined for each scale piece and averaged for each bulb using Eq 6

$$\text{FI}=\frac{\text{Firmness}\;(\text{N})\times \text{Thickness}\;\text{of}\;\text{scale}\;\text{(mm)}}{\text{Deformation}\;\text{at}\;\text{firmness}\;\text{(mm)}}$$ (6)

The dry matter content was determined on the remaining bulb tissue. The scales were cut into smaller pieces, frozen in liquid nitrogen, and freeze-dried in a CRIST 173 dryer (Osterode am Harz, Germany). The dry matter content was determined by weighing the onion pieces before and after freeze-drying and given as a dry matter percentage on a wet weight basis.

### Evaluation of sprouting, rooting and diseases

The external quality and internal quality of the onions were evaluated the day after sampling. Rooting and sprouting, as part of the external quality parameters, were evaluated first, and the bulbs were then cut from the neck to the base with a sterile knife for the disease evaluation. The base plate, bulb neck, and internal and external scales were inspected, and diseases were recorded from their symptoms based on appearance, color and smell, as described by Snowden [4], Schwartz and Mohan [7], and Islam [13]. To verify the diseases, infected tissue was taken with a sterile scalpel and grown on PDA and LB agar plates (Sigma-Alrich Chemie GmbH, Stenheim, Germany) for isolation and characterization of the morphological characteristics [7]. Rooting, sprouting and the incidence of individual diseases were noted for each bulb, and from these numbers, the total incidences of rooting, sprouting and total diseases, and specific diseases were calculated. As bulbs could have more than one disease at a time, the sum of individual diseases could exceed the incidence of total diseases.

### Statistical analysis

The statistical analysis was carried out with R statistical software (Ver. 3.1.0, R Foundation, Vienna, Austria). One- and two-way analysis of variance (ANOVA) tests were applied to identify differences between batches and batches, storage time and batches × storage time. If an interaction was nonsignificant, the model was reduced to a two-way analysis without interaction and further to a one-way analysis. Tukey’s honest significance difference (HSD) test at *P* = 0.05 was applied for multiple comparisons. All measurements were carried out in triplicate. Principal component analysis (PCA) was carried out using the PLS_toolbox under the MATLAB environment (version 9.3.0, MathWorks, Natick, MA, USA) to describe the relations between batches in storage and the bulb weight, bulb size, respiration rate, dry matter content, firmness, and total and individual incidences of disease. To describe the relations between the microclimate and bulb quality (respiration rate and total and individual diseases), a PCA was developed based on data from the last storage time (110 days). The following microclimate data were used in the model: average temperature (T-ave) and average RH (RH-ave) in the middle of the box, sum of the temperature difference between the middle and edge (T-sum), and vapor pressure defect sum (VPD-sum) at the middle of the box. Mean values were used, and the data were mean-centered and auto scaled for the analyses.

## Results and discussion

### Microclimate in drying

**Fig 2:**
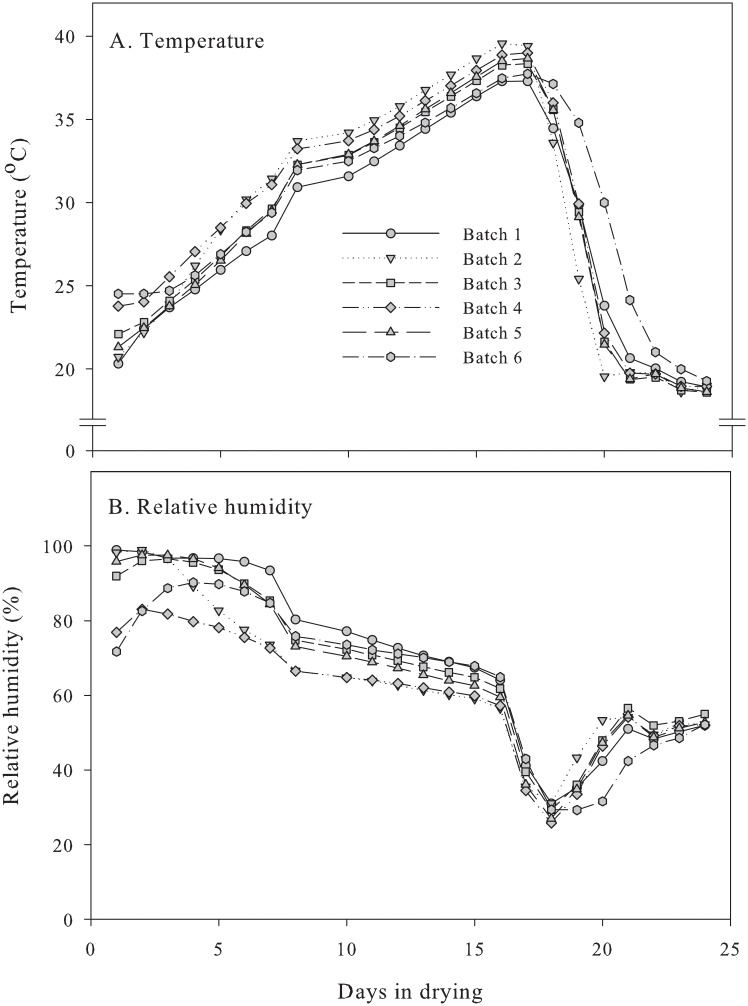
Temperature (A) and relative humidity (B) in the middle of two-ton boxes during drying of onions.

The temperature and RH in the middle of the boxes during drying are shown in Fig 2. The highest temperature was reached after 16 days of drying (Fig 2A), ranging from 37.3 °C (batch 1) to 39.5 °C (batch 2). Then, temperature decreased steeply, reaching 20 °C in approximately 5 days, while the RH decreased from initially 89% RH at beginning of drying to approximately 61% RH after 16 days and to 29% RH by 18 days, with decreasing temperature before it increased to approximately 53% RH at the end of drying (Fig 2B). Some of the bulbs from batch 1, which had the largest fraction of onions above 60 mm (S1 Table), the highest bulb weight (Table 1), and a high RH inside the box at beginning, developed partially translucent scales during drying. This indicated that the temperature was too high, and some of the cells collapsed and liquid moved from the interior into the intercellular spaces [16].

Harrow and Harris [9] noted that the maximum drying temperature for onions is 35 °C for 6 days. However, Schroeder, Humann [17] reported that the maximum drying temperature was 38 °C for 2 days, as higher temperatures for prolonged times would increase softening and enhance bacterial growth in cold storage due to the leaking of nutrients from the cells into the intercellular spaces, causing translucency [16]. During the cool down, the RH inside the boxes reached 25–32% (Fig 2B), and the levels were thus well below 60% RH, which is the recommended minimum level for preventing skin cracking during periods with high temperatures in drying [9, 13]. Skin cracking was not noted in this experiment, but in another with organically grown ‘Hylander’ from the same farm we observed that more onions had white scales that could be seen through the cracked tunic following 14 days drying at 35 °C and 50–52% RH compared to drying at 20 °C and constant 56% RH [13].

**Fig 3:**
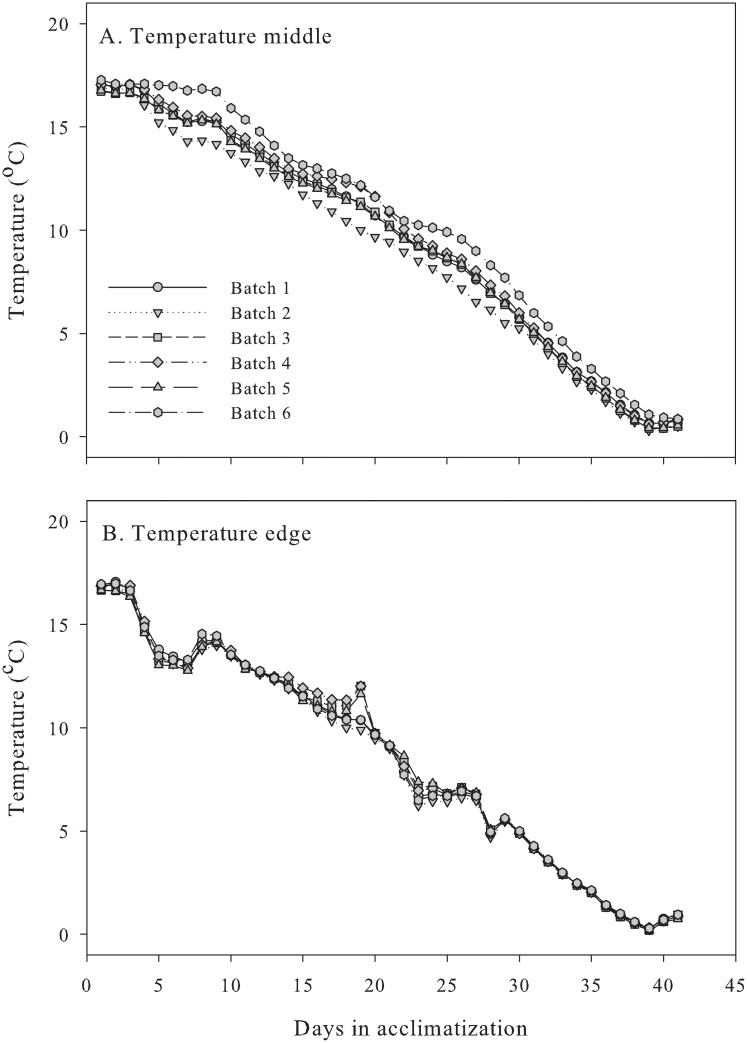
Temperature in the middle (A), and at the edge (B) of two-ton boxes during acclimatization of onions.

During acclimatization, the temperature slowly decreased to cold storage at 0.5 °C both in the middle and at the edge of the boxes (Fig 3). Interestingly, batch 6 responded slower and batch 2 responded faster to the temperature changes (Figs 2A and 3A), although the edge temperatures were similar in all of the boxes (Fig 3B). The direct filling of batch 6 in the field at the hopper most probably reduced the air velocity inside the box [18] because of more foreign material and weeds, which could restrict air exchange and delay cooling. The opposite was noted in batch 2. We did not determine the air velocity or weight percentage of foreign material in the two-ton boxes, but we determined the weight percentage of stones and it varied between 0.3% and 1.7% with the lowest percentage in batch 2 and the highest in batch 3 (S1 Table). These results indicate that the temperature in the vicinity of onions may be influenced by factors other than air temperature in storage and respiration rate [18].

### Differences in microclimate between batches

With the Hortisens sensors, it was possible to follow the online temperature and RH readings in the middle and at the edge of boxes during long-term storage. The microclimate in the middle of the boxes represented the microclimate in the vicinity of the onions, while the microclimate at the edge represented the microclimate of the storage air. The average temperature in the cold storage ranged from 0.30 °C to 0.55 °C at the edge and from 0.39 °C to 0.82 °C in the middle of boxes, while the RH ranged from 89.1% to 91.4% at the edge and from 90.1% to 93.2% in the middle of boxes (Table 2). Overall, the storage temperature and RH in the middle of boxes were higher than at the edge due to metabolic activity and release of respiratory heat and water vapor from the stored onions [13, 19, 20]. The highest average temperature and daily temperature difference sum were observed for batch 1, batch 4 and batch 6 (Table 2). Temperature differences between the boxes at the middle may indicate variation in the metabolic activity of the batches and or restriction in the air velocities inside boxes while differences at the edge may indicate variation in the air temperature of the room and release of heat from bulbs by conductivity [21].

**Table 2:** Microclimate in two-ton boxes with different batches of onions between 8 December 2015 and 27 March 2016.

Batch no.	Average temperature middle (°C)	Average temperature edge (°C)	Daily temperature difference sum^a^ (°C)	Average RH middle (%)	Average RH edge (%)	Vapor pressure deficit sum^a^ (kPa)
Code^b^	T-ave		T-sum	RH-ave		VPD-sum
B1	0.65 ± 0.21^c^	0.50 ± 0.44^c^	18.6	90.4 ± 2.6^c^	90.7 ± 2.5^c^	158
B2	0.39 ± 0.23	0.30 ± 0.42	13.1	90.1 ± 1.9	90.5 ± 1.9	159
B3	0.49 ± 0.22	0.40 ± 0.45	13.8	93.2 ± 1.7	91.4 ± 2.1	113
B4	0.64 ± 0.22	0.41 ± 0.46	21.2	91.0 ± 2.8	89.4 ± 2.1	150
B5	0.47 ± 0.23	0.42 ± 0.41	11.3	91.9 ± 2.3	89.1 ± 1.7	133
B6	0.82 ± 0.19	0.55 ± 0.44	25.4	91.8 ± 2.3	89.1 ± 1.5	136

^a^ See Material and methods for calculations.

^b^ Code used in the PCA.

^c^ Means ± standard error (*N* = 110).

**Fig 4:**
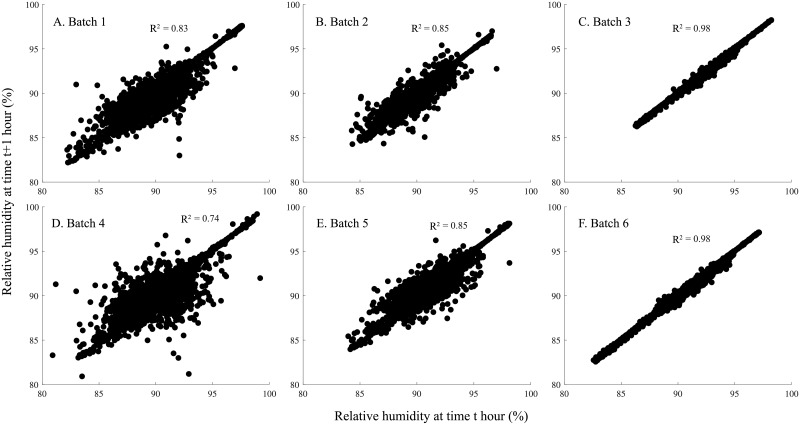
Lag plot of the relative humidity in the middle of two-ton boxes during 110 days of cold storage of the six batches. The correlation coefficient (r) between relative humidity at time *t* and time *t* + 1 hour for each batch is shown on the figures.

As the VACCTEC storage system uses a slight vacuum to create uniform air velocity in storage, it is most likely that the observed differences in the temperature and RH between batches were caused by variation in the bulb metabolic activity rather than differences in air velocity, except for batch 6, which was stored without presorting. Thus, this batch may have experienced a slightly lower air velocity in storage. Overall, the average temperature in storage was low (Table 2), and it prolonged the storability of the onions [1, 22], while humidity was well above the recommended level, which is 65–75% RH [13, 22]. High humidity in storage may increase the risk for condensation of water vapor at the bulb surfaces during periods with variable temperatures, which will promote microbial growth and reduce storability [3, 4]. The lowest average RH during cold storage was observed in batch 2, and the highest was observed in batch 3 (Table 2). More importantly were the variations in the RH during storage, as shown in the lag plot in Fig 4. Overall, the correlation coefficients were high for batch 3 and batch 6 (r = 0.99) showing that there was little variation between consecutive RH measurements and low for batch 4 (r = 0.86) and batch 1 (r = 0.91), showing the opposite result. High variations in consecutive RH measurements as for batch 1 and 4 may lead to periodical condensation and increased microbial growth [4], which is unwanted in onion storage.

**Fig 5:**
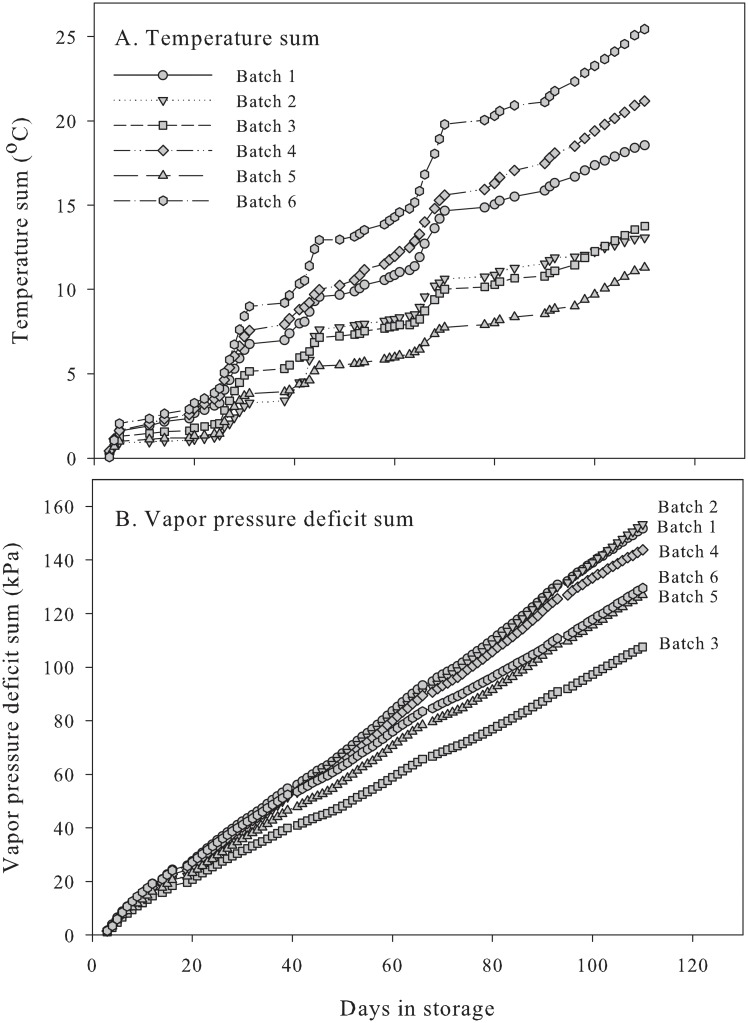
Temperature sum (A) and vapor pressure deficit sum (B) inside two-ton boxes with onions. See Material and methods for calculations.

The daily temperature difference and VPD sum during storage are shown in Fig 5 and Table 2. The highest daily temperature difference sums were calculated for batch 6 (25.4 °C), batch 4 (21.2 °C), and batch 1 (18.6 °C), which also had the highest average batch temperatures (Table 2). As previously noted, batch 6 was stored without presorting, and this may have influenced the air velocity and removal of respiratory heat and thus the storage temperature inside the box. The daily temperature difference sum was lowest in batch 5 (11.3 °C), while it was slightly higher in batch 2 (13.1 °C) and batch 3 (13.8 °C), which had parallel daily temperature difference sum curves (Fig 5A). From the RH readings, a daily VPD sum was calculated for the middle of the boxes (Table 2; Fig 5B). The highest VPD sums were observed for batch 1 and batch 2 (158–159 kPa) and batch 4 (150 kPa), and the lowest was observed for batch 3 (113 kPa). It has been shown that there is a positive relationship between the VPD and evapotranspiration in horticultural crops [23, 24], e.g., a high VPD is related to high evapotranspiration and weight losses in storage [25, 26]. We did not determine weight loss in our study, but previously, we have shown that a high VPD in drying is related to high weight losses [13].

### Differences in quality between batches

**Fig 6:**
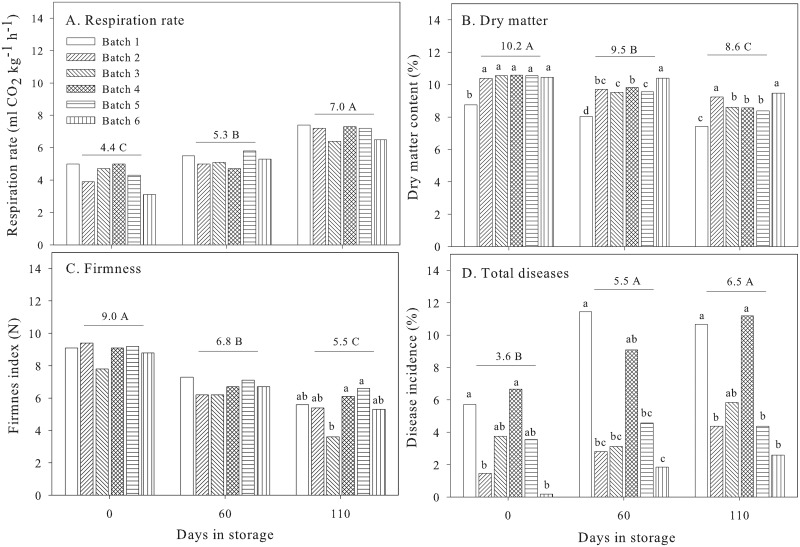
Respiration rate (A), dry matter (B), firmness (C) and total diseases (D) during storage. Means followed by different capital letters within figure, and means followed by different small letters within figure for storage time are significant different at *P* = 0.05 according to Tukey’s honest significance difference test.

The respiration rate, dry matter content and firmness of healthy onions are shown in Fig 6. The average respiration rate was 4.4 ml CO_2_ kg^-1^ h^-1^ at the beginning of cold storage (day 0), increasing to 5.3 ml CO_2_ kg^-1^ h^-1^ at day 60 and further to 7.0 ml CO_2_ kg^-1^ h^-1^ at day 110. There were no significant differences in the respiration rate between the batches at different days (Fig 6A) due to a high variation existing between subsamples within each batch. Increased respiration during the prolonged storage of healthy onions has been previously reported in relation to a break of dormancy [12, 27, 28] and increased activity, which, in the beginning, can be seen inside the bulbs as a growth of secondary roots from the basal plate (rooting) and formation of new shoots from the stem plate (sprouting) [13, 29]. No visual signs of rooting and sprouting were observed during the storage of onions, although the storage humidity was above 90% RH, which promotes rooting [1, 3, 22].

The dry matter content declined in all of the batches during storage (Fig 6B). The average content was 10.2% at the beginning, decreasing to 9.5% at day 60 and to 8.6% at day 110. This result indicates that the dry matter pool, which is used for respiration, decreased in storage regardless of the raw material quality at harvest. Kahsay, Abay [30] showed that the dry matter content of onions decreases with time in storage. Overall, there were significant differences in the dry matter content at harvest and during storage between batches. On average, batch 6 had the highest dry matter content and batch 1 the lowest content, while the contents were intermediate for batches 3, 4 and 5. The relative decrease in the dry matter content also varied between batches (data not shown). Batches 3, 4 and 5 had the highest decreases in dry matter content during 110 days storage (18.5–20.5%), batch 1 had an intermediate decrease (15.2%), and batches 2 and 6 the lowest decreases (9.4–11.1%). In summary, batch 6 had the highest dry matter content and the lowest dry matter decline during storage, and it corresponded to a low disease incidence (Fig 6D and S2 Table).

Overall, the bulb firmness decreased during storage, from 9.0 N at day 0 to 6.8 N at day 60 and further to 5.5 N at day 110 (Fig 6C). Thus, the greatest decrease occurred between day 0 and day 60, which is probably due to changes in cell wall strength and turgor during storage [31]. Many factors influence the turgor pressure of plant cells, e.g., the contents of osmotic solutes in the vacuole, such as sugars, salts, proteins and organic acids and the amount of moisture lost in evapotranspiration [26, 32]. It has been shown that the bulb firmness is positively related to the dry matter content in onions [31] and that bulbs with a higher dry matter content are firmer and store better than those with a lower content [10]. Differences between batches in firmness were observed at the end of storage, and batches 4 and 5 were firmer than batch 3 (Fig 6C).

The average disease incidence increased from 3.6% at day 0 to 5.5% at day 60 and to 6.5% at day 110 (Fig 6D and S2 Table). Moreover, batches varied significantly in their disease incidence in storage (Fig 6D). Overall, batches 1 and 4 had the highest disease incidences throughout storage. Batch 6 the lowest disease incidence, while the other batches had incidences that were in between these (S2 Table). Different diseases and different combinations of diseases were identified from their visual symptoms, color and smell, and morphological characteristics [4, 7]. Gray mold (*Botrytis cinerea*), neck rot (*Botrytis allii* or *B*. *acelada*), blue mold (*Penicillium* spp.), basal rot (*Fusarium* spp.), sour skin (*Burkholderia cepacia*) and slippery skin (*Burkholderia gladioli* pv. *alliicola*) were identified in the onions (S2 Table). Sour skin was the most predominant disease across batches, followed by basal rot and blue mold (S2 Table). Batch 1 had the highest incidence of sour skin (6.7%), followed by batch 4 (3.9%). The lowest incidence of sour skin was observed in batch 6 (0.4%). It is known that onions create optimum growth conditions for sour skin if bulbs are infected in the field and are wounded or improperly dried during curing. Schroeder, Humann [17] reported that the incidence of sour skin increased following drying at 35 °C for 2 days because the onion tissue collapsed during drying, which was also observed in our study at the high drying temperatures (Fig 2A). Batch 4 and batch 1 had the highest basal rot and the highest disease incidence of all of the batches (S1 Table). Batch 4 was infected with basal rot in the field, and symptoms were already visible at harvest (Table 1). In contrast, batch 6 had the lowest disease incidence (S2 Table) and thus the highest storability. The incidence of blue mold did not vary between batches but increased during storage, as the storage humidity generally was high (S2 Table and Table 2). Blue mold is caused by *Penicillium* spp., which is a fungus that sporolates well in lesions and wounds at high humidity and grows readily on fusarium infected tissue, but it may also grow on nonwounded bulbs [3, 7]. Overall, the presence of neck rot and gray mold was neglectable (S2 Table), indicating that the batches were properly dried and sealed during curing [1].

### Marker tools for quality changes in storage

**Fig 7:**
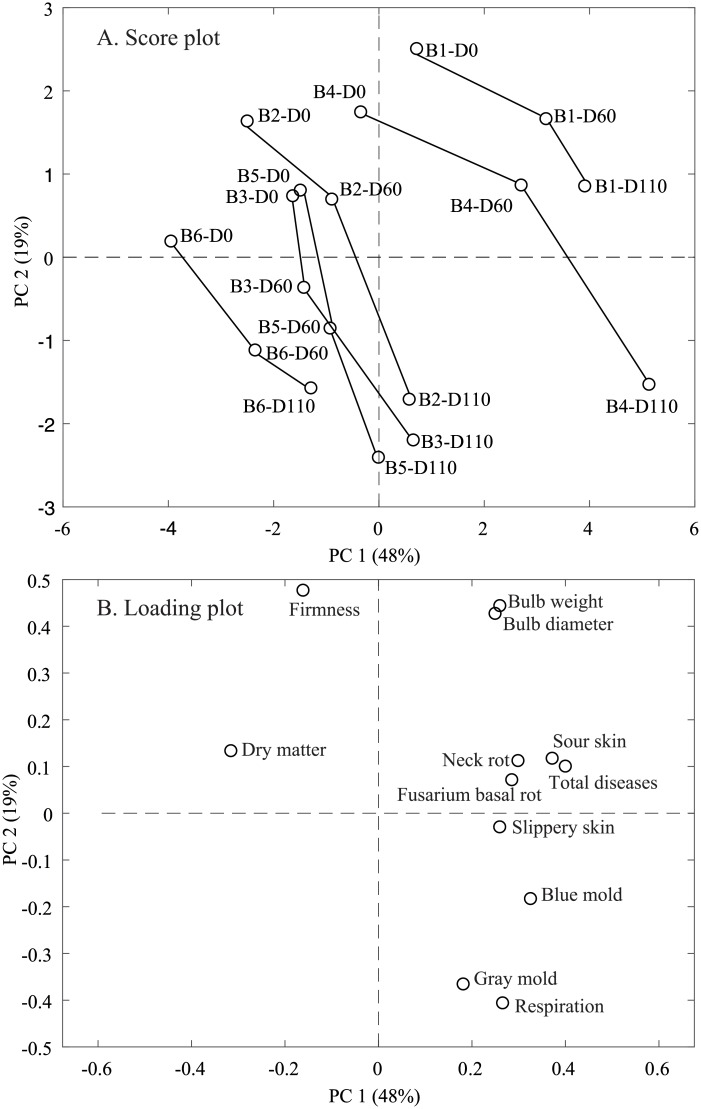
Principal component analysis of bulb quality of six batches (B1 –B6) of onions stored for 0, 60 and 110 days (D). See Table 1 for a description of the batches. Score plot (A). Loading plot (B). The lines connect the different storage times for each batch.

A principal component analysis (PCA) was carried out to explore the relations between the quality of batches in storage and the storage time (Fig 7). This PCA explained 67% of the variation in the data (Fig 7), 48% along principal component (PC) 1 and 19% along PC2. All of the batches were placed in the upper left corner at the beginning, except for batch 1, which was placed in the upper right corner (Fig 7A), as its bulb weight and size was higher and the dry matter content lower than for the other batches (Table 2). When moving in time, the batches moved downwards and mainly to the right in the PCA (Fig 7A), except for batch 6, because the respiration rate and disease incidence increased and the firmness and dry matter content decreased (Figs 6 and 7B). Interestingly, the bulb weight and bulb size were placed on the same side as the total diseases and sour skin and opposite to the dry matter content (Fig 7B), which is in line with the findings that small bulbs with a high dry matter content store better than large bulbs with a low content [10, 33, 34]. Larger bulbs also dry slower and seal necks and tunics later than smaller bulbs as they have more bladed leaves and thicker necks that have to be dried during curing [8, 35]. This may result in inappropriate drying, increased microbial growth and reduced storability of bulbs [4, 7, 8, 24, 30]. We did not inspect the necks and tunic after drying, but we have previously observed that drying at 35 °C for 2 weeks compared to 20 °C produces less bulbs with incomplete dried scales [13]. Therefore, the high sour skin incidence in batch 1 was most likely due to high temperature in curing (Fig 2A) and a high water content at beginning of drying resulting in cell collapse during curing and higher nutrient availability for bacterial growth in storage. Slippery skin, blue mold and gray mold were placed in the lower right quadrant of the loading plot together with the respiration rate (Fig 7B), and this showed that these diseases increased during bulb senescence along with increased respiration rate.

The changes in quality during storage were similar among batch 2 (‘Hylander’), batch 3 (‘Hypark’) and batch 5 (‘Summit’ field 247), as the result moved from the upper left corner downwards and slightly to the right with the storage time (Figs 6 and 7A). In contrast, the bulbs of batch 1 and batch 4 separated from the other batches, as they were heavily diseased at the beginning of storage and continued to develop diseases during storage (Figs 6D and 7A). The bulbs of batch 6 also behaved differently, as it moved slightly downwards but remained on the left side as storage proceeded (Fig 7A), indicating that this batch had a low disease incidence throughout storage (Fig 6D).

**Fig 8:**
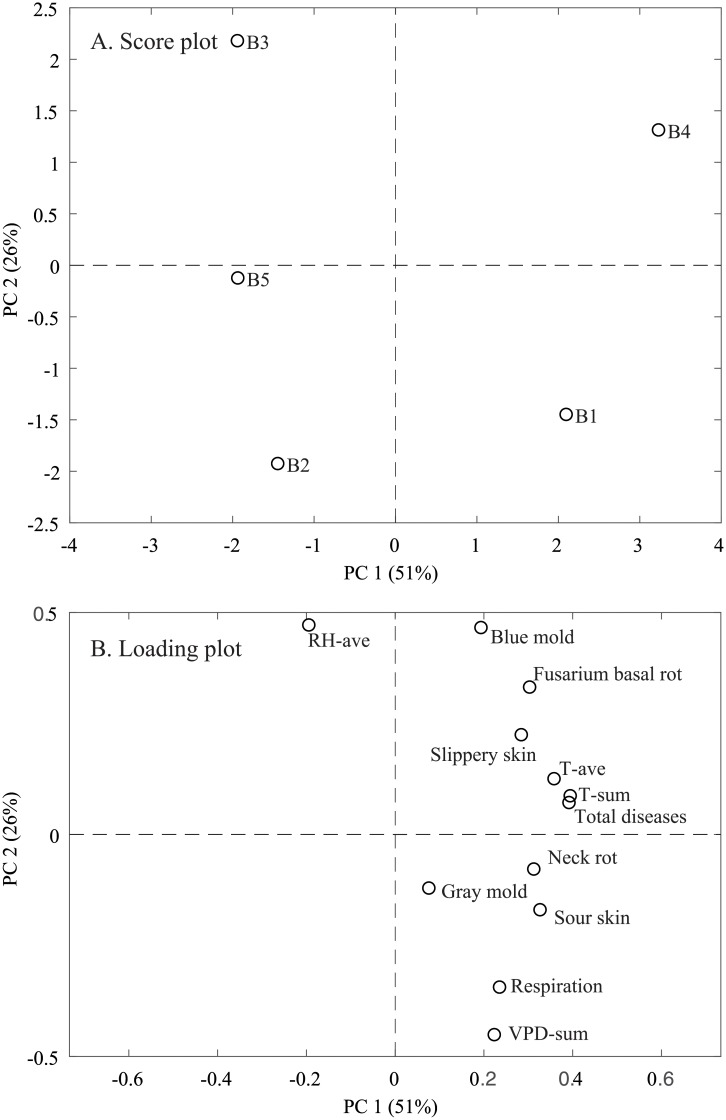
Principal component analysis of microclimate and bulb quality of five batches (B1 –B5) of onions after 110 days of storage. See Table 1 for a description of the batches and Table 2 for abbreviations. Score plot (A). Loading plot (B).

A high storability of batch 6 was unexpected, as it had clear symptoms of *Botrytis* spp. on the leaves in the field (Table 1), which increases the risk for *Botrytis* infection in storage [7]. Moreover, the average temperature in storage and the daily temperature difference sum were higher for batch 6 than they were for the other batches (Table 2), which may indicate an increased metabolic activity in storage. However, batch 6 had the lowest disease incidence of all of the batches (Fig 6D and S2 Table). Therefore, there was no indication that a higher storage temperature was related to an increased metabolic activity in this batch. As stated before, batch 6 behaved differently in storage than the other batches, and this was probably because it was stored without presorting, which restricted ventilation and increased the storage temperature in the vicinity of the onions. A PCA was prepared after 110 days of cold storage without batch 6 to study the relation between the microclimate and the storage quality of the remaining batches (Fig 8). The score plot showed that batch 1 and batch 4 separated from the other batches along PC1, explaining 51% of the variation, and along PC2, explaining 26% of the variation in the data (Fig 8A). Batch 2, batch 3, and batch 5 were placed in the left of the score plot on the same side as the average RH (Fig 8B). In contrast, the average temperature inside the boxes and the daily temperature difference sums were placed near the total diseases (Fig 8B). This indicates that the temperature in the vicinity of onions could be a good indicator for onion quality in storage, e.g., that onions with a low average temperature in storage have a higher storability than onions with a higher average temperature in storage, given that onions are treated similarly prior to filling of boxes and placed in the same storage room.

## Concluding remarks

Online measurements of the microclimate in the vicinity of onions were evaluated as a tool for monitoring the quality changes in onions during storage. The results show that the temperature and relative humidity in the vicinity of the onions varied during drying and reached levels which damaged cells and formed translucent scales and could promote skin cracking. Additionally, temperature varied between batches and the average temperature was higher for batches that had high, rather than low, disease incidence in storage. Moreover, the variation in the RH in storage was higher for batches with high, rather than low, disease incidence. In conclusion, online measurements of temperature and RH in the vicinity of onions is a promising tool for better control of the different processes in commercial onion storage and for identifying onion batches with differences in storability early in storage.

## Supporting information

S1 Table Percentage of stones and size distribution of usable onions in the two-ton boxes. (DOCX)

S2 Table Incidence of diseases (%) during storage of the six onion batches. (DOCX)

S1 Dataset All microclimate and quality data. (XLSX)
