# Supplementary material for: Online measurement of temperature and relative humidity as marker tools for quality changes in onion bulbs during storage
Source: PLoS One. 2019 Jan 17;14(1):e0210577. doi: 10.1371/journal.pone.0210577 (PMC6336314; doi:10.1371/journal.pone.0210577)
Supplement: S1 Table — (DOCX) [file pone.0210577.s001.docx]

**S1 Table. Percentage of stones and size distribution of usable onions in the two-ton boxes.**

| Batch^a^ | Bulb weight^b^ | Stones^c^ |  | Size distribution^d^ (%) | | | |
| --- | --- | --- | --- | --- | --- | --- | --- |
|  | (g) | (%) |  | <40 mm | 40-60 mm | >60-80 mm | >80 mm |
| B1 | 130 | 0.6 |  | 0.4 | 20 | 49 | 31 |
| B2 | 110 | 0.3 |  | 0.2 | 37 | 48 | 14 |
| B3 | 95 | 1.7 |  | 1.0 | 60 | 34 | 4.9 |
| B4 | 110 | 1.1 |  | 0.8 | 52 | 42 | 4.8 |
| B5 | 91 | 1.3 |  | 1.2 | 68 | 28 | 3.1 |
| B6 | 84 | 0.6 |  | 4.2 | 81 | 14 | 1.0 |

^a^ See Table 1 for a description of the batches.

^b^ Bulb weight is taken from Table 1.

^c^ Weight percentage of stones of the total weight of material in the two-ton boxes after storage.

^d^ Weigh ratio of the different size categories of usable onions in the two-ton boxes.
