# Supplementary material for: Online measurement of temperature and relative humidity as marker tools for quality changes in onion bulbs during storage
Source: PLoS One. 2019 Jan 17;14(1):e0210577. doi: 10.1371/journal.pone.0210577 (PMC6336314; doi:10.1371/journal.pone.0210577)
Supplement: S2 Table — (DOCX) [file pone.0210577.s002.docx]

**S2 Table. Incidence of diseases (%) during storage of the six onion batches.^a^**

| Diseases | Gray mold  *(Botrytis cinerea)* | Neck rot  *(Botrytis allii / B. acelada)* | Blue mold *(Penicillium* spp.) | Fusarium basal rot (*Fusarium oxysporum*) | Sour skin (*Burkholderia cepacia*) | Slippery skin (*Burkholderia gladioli* pv. *Allicola*) | Total diseases^b^ |
| --- | --- | --- | --- | --- | --- | --- | --- |
| Batch^c^ |  |  |  |  |  |  |  |
| B1 | 0.1b^d^ | 0.5a | 2.2a | 2.5b | 6.7a | 0.4b | 9.4a |
| B2 | 1.0a | 0.0a | 1.0a | 0.6b | 1.9bc | 0.3b | 2.9b |
| B3 | 0.0b | 0.1a | 1.6a | 1.9b | 2.4bc | 0.3b | 4.2b |
| B4 | 1.0a | 0.6a | 2.8a | 6.6a | 3.9b | 3.0a | 8.9a |
| B5 | 0.8ab | 0.0a | 1.9a | 1.7b | 2.1bc | 0.4b | 4.2b |
| B6 | 0.0b | 0.1a | 0.9a | 0.7b | 0.4c | 0.2b | 1.5b |
| Average over batches | 0.5 | 0.2 | 1.7 | 2.4 | 3.0 | 0.8 | 5.2 |
| Significance level | *P* ≤ 0.05 | ns | ns | *P* ≤ 0.001 | *P* ≤ 0.001 | *P* ≤ 0.001 | *P* ≤ 0.001 |
| Storage day |  |  |  |  |  |  |  |
| 0 | 0.0b | 0.0a | 0.9b | 2.1a | 1.6b | 0.6a | 3.6b |
| 60 | 0.1b | 0.2a | 2.0ab | 2.4a | 3.4a | 0.7a | 5.5a |
| 110 | 1.4a | 0.3a | 2.3b | 2.6a | 3.8a | 1.1a | 6.5a |
| Significance level | *P* ≤ 0.001 | ns | *P* ≤ 0.05 | ns | *P* ≤ 0.01 | ns | *P* ≤ 0.001 |

^a^ All data were analyzed by two-way ANOVA without interaction except for gray mold which showed interaction between batch and storage time. Removing the interaction in the analysis, however, did not change the grey mold results.

^b^ Total diseases is the total number of bulbs in a batch having one or more diseases.

^c^ See Table 1 for a description of batches.

^d^ Means in columns followed by different letters within batch or storage day are significant different at *P* = 0.05 according to Tukey’s honest significance difference test.

ns, not significant.
